# Tunneling Nanotube-like Structures in *Giardia duodenalis*

**DOI:** 10.3390/cells13181538

**Published:** 2024-09-13

**Authors:** Victor Midlej, Albano H. Tenaglia, Hugo D. Luján, Wanderley de Souza

**Affiliations:** 1 Structural Biology Laboratory, Oswaldo Cruz Institution, Fiocruz, Rio de Janeiro 21040-900, Brazil; 2 Consejo Nacional de Investigaciones Científicas y Técnicas (CONICET), Centro de Investigaciones en Química Biológica de Córdoba (CIQUIBIC), Universidad Nacional de Córdoba, Córdoba 5000, Argentina; 3 Departamento de Química Biológica Ranwel Caputto, Facultad de Ciencias Químicas, Universidad Nacional de Córdoba, Córdoba 5000, Argentina; 4 Facultad de Ciencias de la Salud, Universidad Católica de Córdoba (UCC), Córdoba 5004, Argentina; 5 Institute of Medical Microbiology and Hygiene, University Medical Center of the Johannes Gutenberg-University Mainz, 55131 Mainz, Germany; 6 Laboratório de Ultraestrutura Celular Hertha Meyer, Centro de Pesquisa em Medicina de Precisão, Instituto de Biofísica Carlos Chagas Filho, Universidade Federal do Rio de Janeiro, Rio de Janeiro 21941-902, Brazil; wsouza@biof.ufrj.br

**Keywords:** protozoan cell, ultrastructure, tunneling nanotubes, high-resolution microscopy

## Abstract

*Giardia doudenalis* (*lamblia*, *intestinalis*) is a protozoan parasite that inhabits the lumen of the upper small intestine of vertebrates, causing chronic abdominal pains and severe diarrhea, symptoms of giardiasis, a persistent and recurrent infection. This characteristic is mainly due to the presence of membrane variant-specific surface proteins (VSPs) that give this parasite the ability to successively infect the host through antigenic variation. Using high-resolution scanning microscopy (HR-SM), we observed the presence, formation, and extension of tunneling-nanotube-like surface structures in *Giardia*, especially following parasite challenges with VSP antibodies. They were seen all over the parasite surface, both *in vitro* and *in vivo*, showing that *G*. *duodenalis* nanotube formation occurs in complex environments such as the gut. In addition, we also observed that some of these nanotubes displayed a periodic strangulation that produces 100 nm vesicles that seemed to be released in a process similar to that previously observed in *Trypanosoma brucei.* The presence of nanotube-like structures in *G. duodenalis* highlights yet another strategy of cellular communication utilized by these parasites, whether between themselves or with the host cell.

## 1. Introduction

The examination of different cell types via scanning electron microscopy, especially when using high-resolution equipment, has demonstrated the presence of fine projections on the cell surface that differ from the better-known ones, such as the filopodia, lamellopodia, microvilli, etc. [1]. These structures have been called tunneling nanotubes (TNT) or cytonemes, and their general morphology is quite diverse [2]. Actin filaments are generally observed within these projections, as shown in thin sections examined via transmission electron microscopy [3]. More recently, many papers have been published stressing the participation of TNT in direct intercellular communication [3,4,5].

*Giardia* serves as an excellent model for studying cellular mechanisms, particularly due to its streamlined structure and organelles. *Giardia duodenalis* (also known as *G. lamblia* or *G. intestinalis*) is a non-invasive protozoan parasite that infects the upper small intestine, causing giardiasis. Since 2004, giardiasis has been included in the World Health Organization’s Neglected Diseases Initiative [6]. This disease is globally distributed, impacting approximately 280 million people annually [7]. *Giardia* infections can result in irritable bowel syndrome and food allergies, even after the infection has been resolved. Symptoms of giardiasis include watery diarrhea, nausea, epigastric pain, and weight loss [8]. *Giardia* has a straightforward life cycle, consisting of two primary stages: the proliferative trophozoite and the infectious cyst [9]. The parasite is predominantly transmitted through contaminated water, making many developing countries endemic regions [10].

Still, there is little information on TNT-like structures in parasitic protists. Rupp and collaborators [11] described the presence of these structures in the sexual stages of *Plasmodium falciparum* in mosquitoes, especially in activated gametocytes, where these structures establish contact between different cells with lengths varying from 2 to 20 μm. They were observed in both emerging macrogametes and flagellating microgametocytes. Five years later, Szempruch and collaborators [12] showed the presence of membranous nanotube-like structures formed from the flagellar membrane in the posterior region of African trypanosomes and that, subsequently, vesicles were released via the process of strangulation of the tubules.

Using high-resolution scanning microscopy with electrons and ions, Benchimol and colleagues [13] demonstrated the presence of thin surface projections in *Tritrichomonas foetus*, with a mean diameter of 70 nm. Some of them were straightforward, ending freely, with a length that reaching 4 μm. Others seemed to connect adjacent cells. Meanwhile, others showed a non-straight orientation, displaying a curved appearance. They can be seen at the anterior region, in between the anterior flagella, on the tip of the flagella, and in the cell body, especially near the area of attachment of the recurrent flagellum to the cell body. Subsequently, Salas and collaborators [14] reported the presence of similar structures in *Trichomonas vaginalis,* where they protrude from both the surface of the protozoan and the flagella, especially on the attached parasites, and that they connected the parasites. In a recent work on the involvement of antibodies against variable surface antigens in *Giardia duodenalis,* images showed membrane projections emerging from the central flange and the ventral disk [15].

Microscopy, especially scanning electron microscopy (SEM), has been used to analyze the surfaces of many cell types, including pathogenic protozoa [16,17]. More recently, important improvements in SEM have allowed us to obtain high-resolution images in the range of 0.8 to 1 nm. These improvements include (a) the use of field emission guns that generate beams with a small diameter but with variable energy, (b) the use of highly sensitive secondary and backscattered electron detectors well positioned in relation to the sample, and other factors [17]. Also, a new microscope, known as helium ion microscopy (HIM), was developed. This is based on using ions rather than electron beams [18]. This equipment allows the obtainment of high-quality images that can reach a sub-nanometer resolution. Such high resolution is due to the beam’s high brightness using a tiny probe size and the relatively short wavelength of He^+^, enabling the beam to be focused on dimensions as low as 0.3 nm [18].

Here, we describe in more detail the presence of TNT-like structures in *G. duodenalis*, both in axenic cultures and in the complex environment of the gut of experimentally infected gerbils.

## 2. Materials and Methods

### 2.1. Giardia Cultures

*G. duodenalis* assemblage A1 isolate WB (ATCC^®^ 50803, Manassas, VA, USA) clones and the clone GS/M-83 (ATCC^®^ 50581, Manassas, VA, USA) (assemblage B) were cultured in TYI-S33 medium, as previously described [10]. Parasites expressing different VSPs (the clone WB expressing VSP417 and VSP1267 and the clone GS/M-83 expressing VSPH7) were obtained by limiting dilution in 96-well culture plates placed in anaerobic chambers (Anaerogen^TM^ Compact, Thermo Scientific^®^ Oxoid^®^, Cat. # AN0010C, Hampshire, UK) at 37 °C for 5 days, and positive clones were then selected using specific anti-VSP mAb with an immunofluorescence assay (IFA) as described in [10]. Reactive clones were expanded in a culture medium overnight and tested for homogeneity before use [15].

### 2.2. Dynamics of Antibody-Bound VSP Elimination

The induction of nanotube-like structure formation was performed with VSP antibody incubation, as described by Tenaglia and collaborators [15]. Briefly, trophozoites of the clones VSP417, VSP1267, and VSPH7 were treated with 2.5 nM of their cognate anti-VSP mAbs (mAb 7C2, mAb 7F5, and mAb G10/4, respectively) in TYI-S-33 medium on ice for 1 h. Then, cells were washed three times with ice-cold PBS, resuspended in 7 mL of TYI-S-33 medium, and incubated at 37 °C for 0, 15, 30, and 60 min. At each time point, trophozoites were placed on ice for detachment, washed twice with ice-cold filtered PBS, and fixed with 4% FA in sodium phosphate 0.1 M. Immunogold labeling was performed using goat anti-mouse IgG-(H-L)-gold 10 nm (Abcam, Cat. # ab39619, Cambridge, UK), and then the cells were fixed with 2.5% glutaraldehyde in 0.1 M cacodylate buffer (pH 7.2) and processed for high-resolution scanning electron microscopy (HR-SEM) or transmission electron microscopy (TEM).

### 2.3. Animals and Giardia Infections

Gerbils (6–8-week-old) of both sexes were housed in the vivarium of the CIDIE under specific pathogen-free (SPF) conditions in microisolator cages (Techniplast^TM^, Varese, Italy), following the NIH guidelines for laboratory animals. Before infection with *G. duodenalis*, six-week-old gerbils were tested to ensure their negativity for serum antibodies against *Giardia* antigens via ELISA, as previously reported [10]. Infections were carried out through the orogastric administration of 2 × 10^5^ trophozoites of the clone VSP417 resuspended in 500 µL of PBS. Randomly selected gerbils were euthanized in a CO_2_ chamber on days 5, 7, 10, 12, and 14 post infection. The first 14 cm of the upper small intestine, measured from its junction with the stomach, was removed and dissected longitudinally using surgical scissors. The intestine was then divided into two sections. One section was cut into small pieces and fixed in 2.5% glutaraldehyde in 0.1 M cacodylate buffer at pH 7.2. This sample was then processed for electron microscopy as described below. The other section was used to extract and analyze the expression of VSP417 in the trophozoite population. This section of the intestine was briefly incubated in ice-cold PBS for 30 min. The supernatants were collected, Giardia trophozoites were labeled, and VSP characterization was carried out by means of flow cytometry as described.

### 2.4. VSP Characterization by Flow Cytometry

Trophozoites extracted from the intestine were processed for flow cytometry as described in our previous study [10]. Briefly, cells were treated with mAb 7C2 and VSP417^(−)^-pAb in TYI-S-33 medium on ice. After staining, the cells were fixed with 4% PFA and then blocked with ammonium chloride and BSA in PBS. Secondary labeling was performed using goat anti-mouse IgG (H-L)-phycoerythrin, goat anti-rat IgG (H-L)-biotin, and streptavidin-Alexa Fluor™ 488. Flow cytometry analysis was conducted using a BD Accuri C6™ instrument (BD Biosciences^®^, Franklin Lakes, NJ, USA). Data analysis and graph generation were performed using FlowJo™ 7.6 software (TreeStar^®^, Ashland, OR, USA).

### 2.5. High-Resolution Scanning Electron Microscopy (HR-SEM) and Helium Ion Microscopy (HIM)

Briefly, glutaraldehyde-fixed cells were post-fixed in 1% OsO4 for 15 min. Then, samples were dehydrated in a crescent series of ethanol up to 100%, critical point-dried with liquid CO_2_. For high-resolution scanning microscopy (HR-SEM) analysis, the sputter coating step was performed using a thin layer (2 nm) of platinum, and cells were observed on an Auriga^TM^ HR-SEM (Carl Zeiss, Oberkochen, Germany). For HIM, cell samples were processed similarly to those used for HR-SEM, except for the ion-sputter step (i.e., the cells were not coated). The images were captured using a 28 kV ion energy, a working distance of 8.5 mm, and a Blanker current of 1.6 pA on an ORION HIM (Carl Zeiss, Oberkochen, Germany). No conductive coatings were applied to the samples before imaging to preserve the sample surface information. The images were formed from detecting induced secondary electrons and constructed as a 2048–2240-pixel array. Samples were transferred into the HIM via a load–lock system and were maintained at a vacuum of 2–3 × 10^−7^ Torr during the imaging session. Charge control was maintained using a low-energy electron flood gun, which was applied in a temporally interlaced manner with the imaging beam. Images were formed by collecting the secondary electrons elicited by the interaction between the helium ion beam and the sample with an Everhart–Thornley microchannel plate [19]. A wideband detector was used to detect micro-ampere low-energy electron currents. This detector is also widely used in SEM and consists of a scintillator placed inside a Faraday cage that draws the elicited low-voltage secondary electrons toward it. The resulting photons are collected, turned into electrons, and amplified by a photomultiplier tube (PMT). The PMT signal is then digitized using an A/D converter and displayed as a gray value in each pixel of the resulting image. The scanning of the helium ion beam and the image’s formation are synchronized so that there is a corresponding signal or gray value in the resulting image for any given coordinate. No post-processing procedures were applied to the digital images besides brightness and contrast adjustment. The image signal was acquired in a line-averaging mode, with either 32 or 64 lines integrated into each line in the final image. Charge neutralization was applied after each line pass of the beam.

### 2.6. Transmission Electron Microscopy (TEM)

Glutaraldehyde-fixed cells were post-fixed with 1% OsO4 and 0.8% potassium ferrocyanide for 40 min. The samples were dehydrated in crescent grades of acetone up to 100% and embedded in epoxy resin. Ultrathin sections (50–60 nm thick) were cut, collected, and stained with uranyl acetate and lead citrate. Lastly, the samples were analyzed using a Tecnai^TM^ Spirit TEM (FEI Co., Hillsboro, OR, USA).

## 3. Results and Discussion

**Figure 1:**
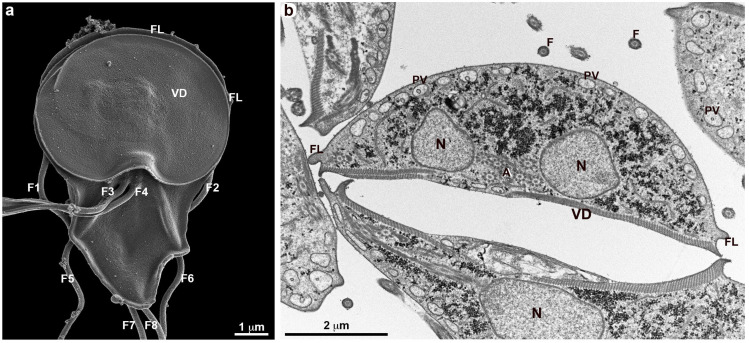
Overview of the ultrastructure of *G. duodenalis* trophozoites. VSPH7 clones were stimulated with anti-H7 antibodies and immediately fixed. (**a**) The ventral region of a trophozoite observed by HR-SEM shows that the parasite’s membrane does not present extensions. The folds in the posterior region above the ventral disk (VD) correspond to the flange (FL). The four pairs of flagella (F1–F8) are visible. (**b**) The parasite observed by TEM reveals the two nuclei (N), peripheral vesicles (PV), the ventral disk (VD), and axonemes (A). The plasma membrane appears to be continuous, with a homogeneous anti-H7 labeling along its length.

**Figure 2:**
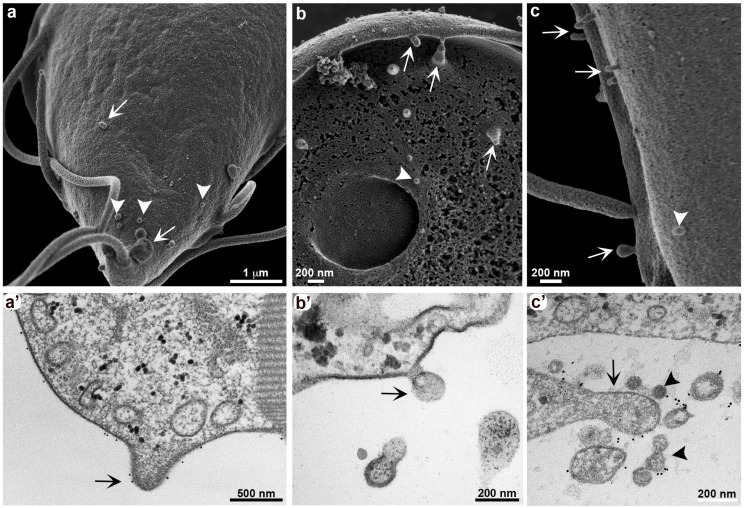
Membrane protrusion and vesicle formation in *G. duodenalis*. Different cell clones were stimulated for 30 min with their respective antibodies before fixation for electron microscopy. Both HR-SEM and TEM show that all clones exhibit plasma membrane protrusions (arrows). Small vesicles can also be observed (arrowheads). Anti-VSP antibody labeling is observed on these membrane protrusions and vesicles by TEM (**a’**–**c’**). (**a**,**a’**) Clone VSP 1267 stimulated with mAb 7F5; (**b**,**b’**) clone VSPH7 stimulated with mAb G10/4; (**c**,**c’**) clone VSP417 stimulated with mAb 7C2.

**Figure 3:**
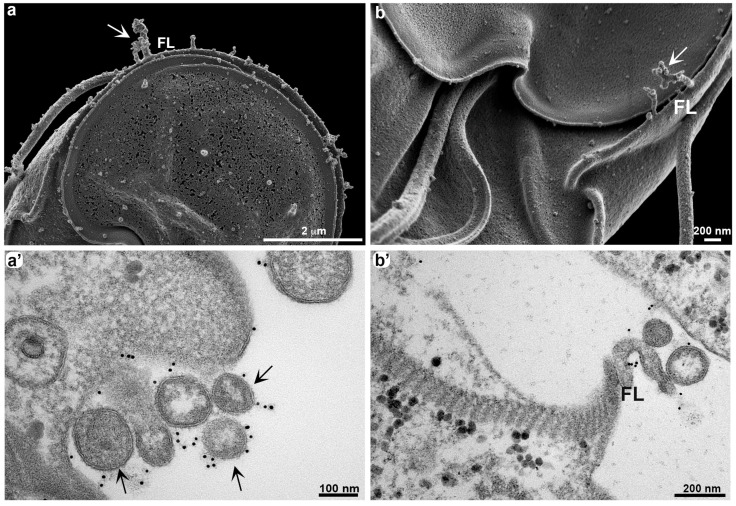
Growth of TNT-like structures formed by extracellular vesicles. *G. duodenalis* clones VSPH7 (**a**,**a’**) and VSP417 (**b**,**b’**) were stimulated with their respective antibodies (mAb G10/4 and mAb 7C2) for 60 min before fixation for electron microscopy. HR-SEM (**a**,**b**) shows an increase in membrane projections in the flange region (FL), where a cluster of vesicles can be observed (arrows). This cluster of disorganized vesicles can be visualized by TEM (**a’**,**b’**). Note that in (**b’**), the vesicles are located in the flange (FL). There is specific anti-VSP antibody labeling in the vesicle accumulation region (**a’**,**b’**).

**Figure 4:**
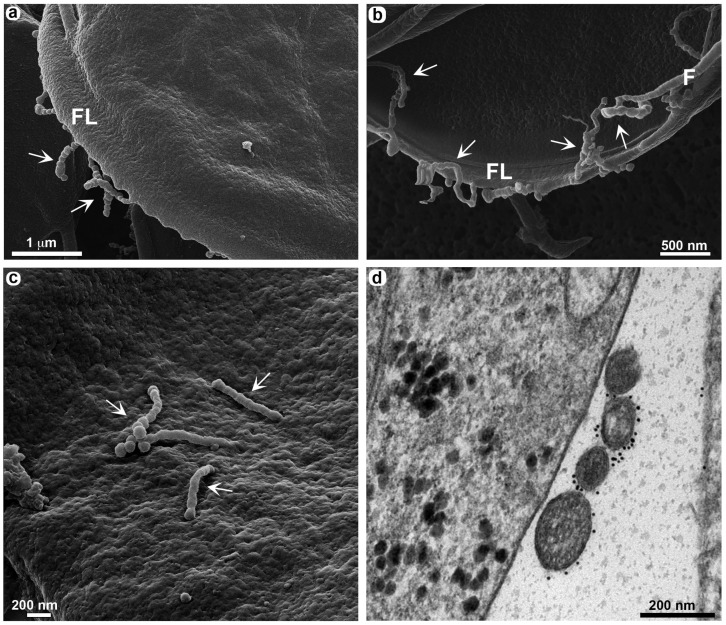
Elongation of TNT-like structures through the sequential organization of vesicles. VSP417 clone parasites were obtained from experimental infection in gerbils at 12 days post infection and observed by HIM (**a**–**c**). Note that a long extension of aligned vesicles (arrows) is seen in the flange (FL) (**a**,**b**), on the flagellum (F) (**b**), and in the dorsal region of the parasite (**c**). (**d**) shows an alignment of vesicles by TEM in a VSP417 clone parasite stimulated *in vitro* for 120 min with mAb 7C2, where intense labeling is seen. In all images, the vesicles can be clearly visualized.

Scanning and transmission electron microscopy have been intensely used by several groups to analyze the structural organization of *G. duodenalis* [17,20,21,22]. In all studies, the surface of trophozoites obtained from axenic cultures (*in vitro*) showed a smooth appearance without any type of surface projections. Even when a high-resolution SEM is used, most of the time, the parasite surface appears, as shown in Figure 1, where small protuberances can be seen, as well as some clumps of small vesicles. With TEM, no protrusions are observed (Figure 1b). In this work, we used *G. duodenalis* incubated with anti-VSP monoclonal antibodies in *in vitro* experiments. For TEM, the parasite was incubated with an antibody against VSPH7 and immediately fixed. There was a homogeneous distribution on the membrane (Figure 1b). However, when the parasites were incubated with an anti-VSP antibody and a more careful analysis was carried out, it was possible to visualize protrusions that appeared initially with a mean diameter of 150 nm and a variable height (Figure 2, Figure 3 and Figure 4).

### 3.1. TNT-like Structures Origin

As demonstrated by Tenaglia and collaborators [15], the parasites can exchange VSPs on the plasma membrane, excluding one type of VSP, by forming extracellular vesicles on the membrane itself after incubation with anti-VSP antibodies. The incubation time with the antibodies favored the formation of projections on the membrane of the parasites *in vitro* (Figure 2). After 30 min of incubation, there was an accumulation of vesicles in a grape cluster-like arrangement [15]. At the same time, plasma membrane projections were noted in the dorsal region (Figure 2a,c), ventral region (Figure 2b), and on the flagella (Figure 2c) of the cell by HR-SEM. We observed the same growth pattern of the projections through TEM (Figure 2a’,c’); note that these regions show the presence of labeling with anti-VSP antibodies. Hemphill and collaborators [23] also used the mAb G10/4 on *G. duodenalis* H7 clones and observed vesicle formation after 5 min of incubation with the antibody using transmission electron microscopy. They suggested that these vesicles were associated with membrane disintegration or “weakening”, potentially contributing to the cytotoxicity of anti-VSP mAbs. In contrast, our observations, along with those of Tenaglia and colleagues [15], indicate that these vesicles are not a sign of cellular damage or death. Instead, they represent a process known as antigen removal coupled to switching (ARCS), where microvesicles selectively remove surface-bound antibodies while simultaneously inducing VSP switching [15].

### 3.2. TNT-like Structures Growth

The growth of thin membrane projections occurs due to the accumulation of vesicles (Figure 3). After 60 min of *G. duodenalis* exposure to anti-VSP antibodies *in vitro*, we observed a greater accumulation of extracellular vesicles (Figure 3a,b), which appear disorganized (Figure 3a’,b’).

It was observed that a similar process occurred *in vivo* when gerbils (*Meriones unguiculatus*) were infected with *Giardia* clones expressing VSP417 [15]. At the onset of the humoral immune response, large membrane extensions and vesicles identical to those observed after *in vitro* treatment with antibodies were seen in trophozoites collected from the small intestine 12 days post infection. Here, we observed these large membrane extensions in more detail (Figure 4). Occasionally, it was possible to see that some of these parasite membrane extensions, called TNT-like structures, display periodic strangulation (Figure 4a–c). Through TEM analysis, we observed an organization of extracellular vesicles after 120 min of exposure of *G. duodenalis* to anti-VSP antibodies *in vitro*, which may suggest that the constriction of the TNT-Like structures is associated with the aligned vesicles (Figure 4d). A similar profile of extracellular vesicle release was observed in *Trypanosoma brucei* [12]. However, the authors demonstrated that these vesicles are released from a formation via membrane nanotubes carrying virulence factors, facilitating resistance to the innate immune response. These data are consistent with those provided by Tenaglia and collaborators [15], but we propose here that some of these vesicles may remain together, forming TNT-like structures. This hypothesis is based on our observations during late stages of post-infection *in vivo* assays, where the constricted profiles seen in Figure 4 were no longer evident. At this time, we do not believe that we are dealing with ECVs, but rather with membrane protrusions resembling the TNT structures observed in other cells. However, we cannot exclude the possibility that VSP switching *in vivo* may exhibit similar characteristics to those observed *in vitro*. In our most recent publication [15], we demonstrated that VSP replacement occurs via vesicle release, but these vesicles are discharged in clusters. Utilizing an actin marker would be crucial for distinguishing the formation of these structures, although conducting such an analysis *in vivo* would be challenging.

Therefore, we cannot exclude the possibility of two types of membrane arrangement: ECVs and TNT-like structures. Additionally, *Giardia* does not have an organized actin filament cytoskeleton as observed in other cells [24]; instead, it exhibits a globular actin organization, similar to what we have observed in the related protozoan *T. vaginalis* [25].

### 3.3. TNT-like Structures Are Present during In Vivo Infection

**Figure 5:**
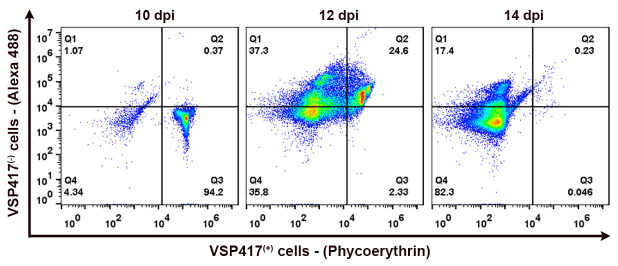
Representative cytograms showing the distribution shift from VSP417^(+)^ to VSP417^(−)^ populations during animal infections with a VSP417 clone. Quadrants were set to reflect positive staining for VSP417 (VSP417^(+)^, horizontal axis), positive staining for VSPs other than VSP417 (VSP417^(−)^, vertical axis), and positivity for both dyes. Density plots indicate the percentage of each population at 10, 12, and 14 days post infection (dpi).

**Figure 6:**
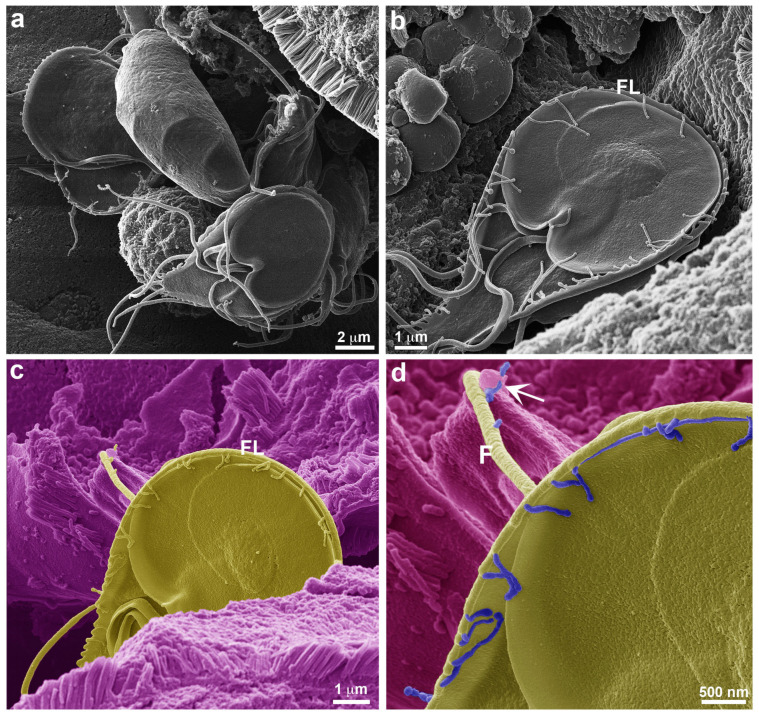
Presence of TNT-like structures during *in vivo* infection. VSP417 clone parasites are observed by HIM 12 days post infection in gerbils. (**a**) Overview of parasites adhered to the intestinal epithelium. (**b**) TNT-like structures can be observed in all regions of the parasite, with a preference for the flange (FL). (**c**) Intimate contact of a TNT-like structure with the intestinal villi; this image is artificially colored to highlight the parasite in yellow and the villi in purple. This relationship can be seen in greater detail in (**d**), where the TNT-like structures are highlighted in blue, showing their projection on the flagellum (F) and contact with the villi (arrow).

**Figure 7:**
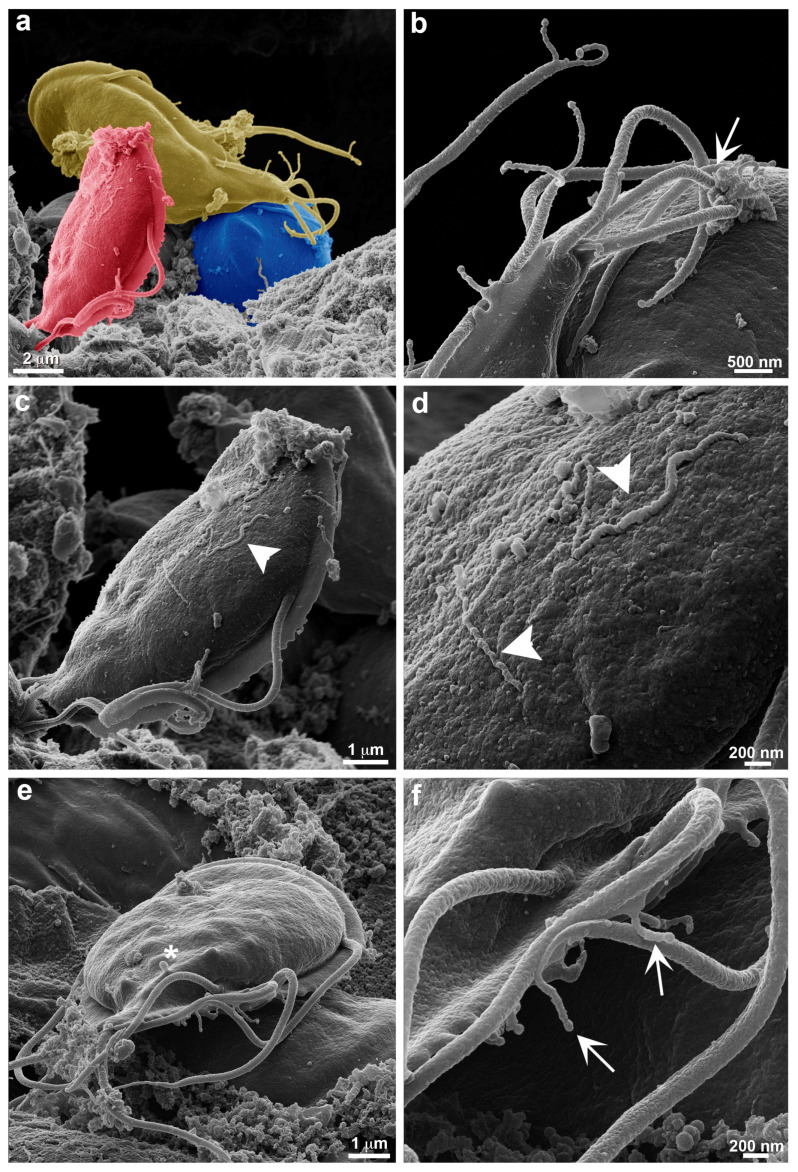
Large extensions of TNT-like structures during *in vivo* infection. VSP417 clone parasites are observed by HIM 14 days post infection in gerbils. (**a**) Interaction between 3 artificially colored parasites for better visualization is observed in the intestine, each parasite is marked with a different color. (**b**) Contact is observed between TNT-like structures emerging from a flagellum (arrow) and the TNT-like structures of an adjacent parasite. (**c**,**d**) Large extensions of TNT-like structures (arrowhead) are observed in the dorsal region of *G. duodenalis*; these TNT-like structures can measure up to 4 µm in length. (**e**) Note that small vesicles can still form in the dorsal region (asterisks), as seen in the *in vitro* analyses. (**f**) TNT-like structures can emerge from any region of the flagella (arrows).

The presence of TNT-like structures is well evidenced *in vivo*. Cytometric analysis of VSP expression in *Giardia* populations within the gut of gerbils (Figure 5) showed that antigenic variation occurs by day 12 post infection. At days 5, 7, and 10 post infection, over 94% of the population still expressed VSP417 at the time of euthanization, indicating that no specific antibodies were yet secreted in the gerbil’s gut. By day 14 post infection, however, no trophozoites expressed the original VSP417, indicating a complete antigenic switch (Figure 5). The cytometry data align with the morphological analyses observed through high-resolution microscopy of the *in vivo* infection. Since the antigenic switching occurs at 12 dpi and peaks at 14 dpi, there is a clear change in the phenotype observed in the TNT-like structures after 12 dpi. The parasites observed in the intestinal lumen of gerbils after 12–14 days post infection exhibit numerous nanotubes, some of which are notably extensive (Figure 6 and Figure 7). The characteristic periodic constrictions in the membrane extensions, commonly seen in earlier stages, are now observed much less frequently. Instead, the nanotubes present a more continuous and elongated projection (Figure 6b,d). TNT-like structures can be observed in all regions of the parasite, with a preference for the flange (Figure 6a,b). During the *in vivo* infection, we observed the intense interaction of *G. duodenalis* with the intestinal epithelial cells (Figure 6). We noticed a specific contact between the TNT-like structures of the parasites and the intestinal microvilli (Figure 6c,d). Mechanisms of interaction between the TNT-like structures and cytonemes of parasites and host cells have been reported [14,26]. It has been shown that contact between *Leishmania donovani* and immune cells, such as B cells and macrophages, stimulates the formation of TNT-like structures in these immune cells, facilitating parasite transmission between them [26]. The results of this study show that parasites can slide between the TNT-like structures of a macrophage to B cells and between B cells. This indicates that during *in vivo* infection with *L. donovani*, the transmission of parasites from infected macrophages to B cells and then their dissemination among B cells, leading to the activation of polyclonal B cells, may occur [26]. It has been demonstrated in *T. vaginalis* that cytonemes of these parasites are in intimate contact with host cells and that such membrane extensions might be related to increased adhesion of the parasite to the host cell, as *Trichomonas* is an extracellular parasite and adhesion is a crucial event in its biology [14]. Other studies with *T. vaginalis* and *T. foetus* show that membrane projections are important in contact with host cells and in establishing infection [27,28]. These results support our observation of the importance of contact between the TNT-like structures of *G. duodenalis* and host cells.

In the *in vivo* analyses, a proximity of parasites to each other is observed when adhering to intestinal cells (Figure 7). Intimate contact between TNT-like structures emerging from the flagella of one parasite is seen with membrane extensions of an adjacent parasite (Figure 7a,b). This may suggest that these structures could facilitate information exchange between *G. duodenalis* populations. In *T. vaginalis*, it was observed that contact between different strains of the parasite through their cytonemes contributed to a greater formation of cellular aggregates [14]. There are several terms used to describe membrane protrusions involved in cell–cell contact. Two types of membrane protrusions that may be confused are cytonemes and tunneling nanotubes (TNTs). The main difference between them is that cytonemes are extremely thin, and their interaction with another cell ends with the membranes coming into close proximity [4]. In contrast, TNTs can exhibit vesicular trafficking and, upon coming into contact with another cell, fuse their membranes to connect the cytoplasm of both cells [29]. However, various organizations of TNT-like structures can be observed, where membrane fusion and cytoplasmic connection may not necessarily occur. This depends on the cell type and the cellular context [30]. This variety of similar structures within the category of membrane protrusions has led to the use of several acceptable terms: thin membrane protrusions, membrane extensions, TNT-like protrusions, cellular bridges, specialized filopodia, and signaling filopodia [30]. In this context, we include the TNT-like structures observed in *G. duodenalis*. A similar role of communication between the same cell types through nanotubes was observed in *T. brucei*, where membrane extensions in the form of nanotubes trigger the release of extracellular vesicles carrying the serum resistance-associated protein necessary for human infectivity from one *Trypanosoma* to another [12]. 

Moreover, the examination of trophozoites within the intestinal lumen of gerbils showed a larger number of TNT-like structures, some of which are very extensive and can reach up to 4 µm in length (Figure 7c,d). TNTs were seen at the distal portion of the flagella (Figure 7c–e) and show the formation of small vesicles as described above for trophozoites obtained from axenic cultures (Figure 7e,f). As shown in *T. foetus*, TNT-like structures can emerge from different portions of the parasite’s cell body [13].

## 4. Conclusions

Based on the characteristics of TNT-like structures found in *G. duodenalis*, particularly their origin and dimensions, we consider that such structures exist in this parasite. Furthermore, they may be either simple or branched and can end freely or be connected between parasites or with host cells. These data open up new perspectives for investigating the molecular mechanisms underlying the formation of TNT-like structures in *Giardia*, particularly regarding the involvement of actin or other molecules in this process.

## Data Availability

This paper provides source data. All other data are available in the main text.
